# Monogalactosyldiacylglycerol synthesis in the outer envelope membrane of chloroplasts is required for enhanced growth under sucrose supplementation

**DOI:** 10.3389/fpls.2014.00280

**Published:** 2014-06-23

**Authors:** Masato Murakawa, Mie Shimojima, Yuichi Shimomura, Koichi Kobayashi, Koichiro Awai, Hiroyuki Ohta

**Affiliations:** ^1^Graduate School of Biological Sciences, Tokyo Institute of TechnologyYokohama, Japan; ^2^Center for Biological Resources and Informatics, Tokyo Institute of TechnologyYokohama, Japan; ^3^Graduate School of Arts and Sciences, Tokyo UniversityTokyo, Japan; ^4^Graduate School of Science, Shizuoka UniversityShizuoka, Japan; ^5^JST PRESTTokyo, Japan; ^6^Earth-Life Science Institute, Tokyo Institute of TechnologyTokyo, Japan; ^7^JST CRESTTokyo, Japan

**Keywords:** galactolipid, monogalactosyldiacylglycerol, MGDG, phosphate deficiency, sucrose

## Abstract

Plant galactolipid synthesis on the outer envelope membranes of chloroplasts is an important biosynthetic pathway for sustained growth under conditions of phosphate (Pi) depletion. During Pi starvation, the amount of digalactosyldiacylglycerol (DGDG) is increased to substitute for the phospholipids that are degraded for supplying Pi. An increase in DGDG concentration depends on an adequate supply of monogalactosyldiacylglycerol (MGDG), which is a substrate for DGDG synthesis and is synthesized by a type-B MGDG synthase, MGD3. Recently, sucrose was suggested to be a global regulator of plant responses to Pi starvation. Thus, we analyzed expression levels of several genes involved in lipid remodeling during Pi starvation in *Arabidopsis thaliana* and found that the abundance of *MGD3* mRNA increased when sucrose was exogenously supplied to the growth medium. Sucrose supplementation retarded the growth of the *Arabidopsis* MGD3 knockout mutant *mgd3* but enhanced the growth of transgenic *Arabidopsis* plants overexpressing MGD3 compared with wild type, indicating the involvement of MGD3 in plant growth under sucrose-replete conditions. Although most features such as chlorophyll content, photosynthetic activity, and Pi content were comparable between wild-type and the transgenic plants overexpressing MGD3, sucrose content in shoot tissues decreased and incorporation of exogenously supplied carbon to DGDG was enhanced in the MGD3-overexpressing plants compared with wild type. Our results suggest that MGD3 plays an important role in supplying DGDG as a component of extraplastidial membranes to support enhanced plant growth under conditions of carbon excess.

## Introduction

Phosphate (Pi) depletion is a serious problem for plant growth worldwide (Lynch, 2011; Kochian, 2012). Among several defense mechanisms that plants use to survive under conditions of Pi depletion, membrane lipid remodeling is common for various plants (Andersson et al., 2003, 2005; Gaude et al., 2004; Jouhet et al., 2004; Benning and Ohta, 2005; Russo et al., 2007; Tjellström et al., 2008; Lambers et al., 2012; Shimojima et al., 2013). In plant cells, ~50% of membrane lipids are composed of galactolipids, which are distinct from biological membranes in mammalian cells (Block et al., 1983; Joyard et al., 1998). Galactolipids are synthesized in chloroplasts and are predominantly located in the thylakoid membranes of chloroplasts under normal growth conditions (Block et al., 1983; Joyard et al., 1998). However, under Pi depletion, phospholipids are degraded to supply Pi for other essential biological processes, whereas galactolipids substitute for phospholipids in extraplastidial membranes (Essigmann et al., 1998; Härtel and Benning, 2000; Andersson et al., 2003, 2005; Jouhet et al., 2004; Nakamura, 2013).

Plants have two major types of galactolipids, namely monogalactosyldiacylglycerol (MGDG) and digalactosyldiacylglycerol (DGDG). MGDG and DGDG constitute ~50 and ~30% of chloroplast membrane lipids, respectively (Block et al., 1983; Joyard et al., 1998). These galactolipids are synthesized in the chloroplast envelope membrane (Douce, 1974). The type-A MGDG synthase, MGD1, in *Arabidopsis thaliana* (*At*MGD1) localizes on the inner envelope membrane and catalyzes the bulk of MGDG synthesis in chloroplasts (Awai et al., 2001; Xu et al., 2005). MGD1 is essential for MGDG synthesis and subsequent DGDG synthesis under normal growth conditions. Indeed, a knockdown mutant of *AtMGD1*, *mgd1-1*, exhibits lower chlorophyll content and photosynthetic activity compared with wild type (WT) and has a defect in thylakoid membrane development (Jarvis et al., 2000; Aronsson et al., 2008). Moreover, a knockout mutant of *AtMGD1*, *mgd1-2*, in which MGDG is decreased by 98% compared with WT, shows a severe defect in embryogenesis shows a severe defect in both embryogenesis and thylakoid membrane development (Kobayashi et al., 2007, 2012).

The other isoforms of MGDG synthase, the type-B MGDG synthases of *Arabidopsis*, namely *At*MGD2 and *At*MGD3, localize on the outer envelope membrane (Awai et al., 2001). Genes for type-B MGDG synthases in *Arabidopsis* were first discovered as the paralogs of *AtMGD1* (Awai et al., 2001). Under nutrient-replete growth conditions, expression of genes encoding type-B MGDG synthase is very low in vegetative tissues but is markedly upregulated during Pi starvation (Awai et al., 2001; Kobayashi et al., 2004). Unlike the case of the *AtMGD1* knockout mutant, the single-knockout mutant of *MGD2* or *MGD3* and the double-knockout mutant do not show a decrease in MGDG or DGDG production or any other particular phenotype different from WT plants grown under normal growth conditions (Kobayashi et al., 2009a). However, the *Arabidopsis mgd3* and *mgd2mgd3* mutants display severe growth retardation and a decrease in DGDG content under Pi depletion, clearly indicating that MGD3-mediated MGDG synthesis has an essential role in survival when Pi is scarce (Kobayashi et al., 2009a). Indeed, recent comprehensive phylogenetic analyses of genes that encode type-B MGDG synthases indicated that the family members are widely distributed in seed plants, suggesting that these genes might have been essential for plants to adapt to Pi deficiency (Kobayashi et al., 2009b; Ohta et al., 2012; Yuzawa et al., 2012). MGDG produced by type-B MGDG synthases is sequentially supplied as a substrate to the DGDG synthases, DGD1 and DGD2 (Härtel et al., 2000; Kelly and Dörmann, 2002; Kelly et al., 2003). The bulk of DGDG produced by galactolipid synthesis via type-B MGDG synthases is transferred to and accumulates in extraplastidial membranes, such as vacuoles, mitochondria, and the plasma membrane, and the presence of DGDG can substitute for phosphatidylcholines (PCs) (Essigmann et al., 1998; Härtel and Benning, 2000; Andersson et al., 2003, 2005; Jouhet et al., 2004; Nakamura, 2013). The sequential events of galactolipid synthesis mainly occur on the outer envelope membranes of chloroplasts where the three enzymes MGD2, MGD3, and DGD2 are localized. Galactolipid synthesis in the outer envelope membrane may be advantageous for transferring DGDG to extraplastidial membranes at the site of contact (Jouhet et al., 2004).

The plant response to exogenously supplied sucrose is similar to its response to Pi starvation. For example, several genes encoding enzymes in carbohydrate metabolism are transcriptionally regulated by Pi content in plants (Nielsen et al., 1998; Ciereszko et al., 2001a,b), and the expression level of a Pi transporter is increased upon supplementation with sucrose (Lejay et al., 2003). Transcript profiling and expression analyses have revealed that the expression of many genes involved in sucrose metabolism is affected by Pi starvation, suggesting that there are interactions between Pi- and sugar-dependent gene regulation (Hammond et al., 2003; Vance et al., 2003; Wu et al., 2003; Misson et al., 2005; Müller et al., 2005, 2007; Hammond and White, 2008). The *Arabidopsis hypersensitive to phosphate starvation1* (*hsp1*) mutant, which overexpresses the sucrose transporter gene, *SUC2*, shows a Pi-starvation-like phenotype even under Pi sufficiency (Lei et al., 2011). Lei et al. (2011) showed that *MGD3* was one of the genes which expression levels were increased in *hsp1* mutant. Although the balance between carbon and Pi content in plant tissues is likely to be important for plant growth, the details remain unknown. Indeed, we have not tested if sucrose supplementation to the growth medium could affect Pi-starvation induced MGDG synthesis. MGDG synthesis on the outer envelope membrane in chloroplasts is known to be important for supplying DGDG, which can substitute for PC in the extraplastidial membranes. Meanwhile, plant growth is known to be enhanced when sucrose is supplied to the growth medium, but the mechanism has been not fully unraveled. The aim of this study was to show the possibility that increased supply of DGDG via upregulation of type-B MGDG synthesis by exogenously supplied sucrose and export of DGDG to the extraplastidial membranes could be partially involved in the enhanced growth when sucrose was supplied to plants. Here we produced *Arabidopsis* transgenic plants overexpressing *AtMGD3* and analyzed the effect of MGDG overproduction on the function of the chloroplast outer envelope membrane under normal growth conditions with or without sucrose supplementation.

## Results

### Expression of *MGD2* and *MGD3* increases upon sucrose supplementation

**Figure 1:**
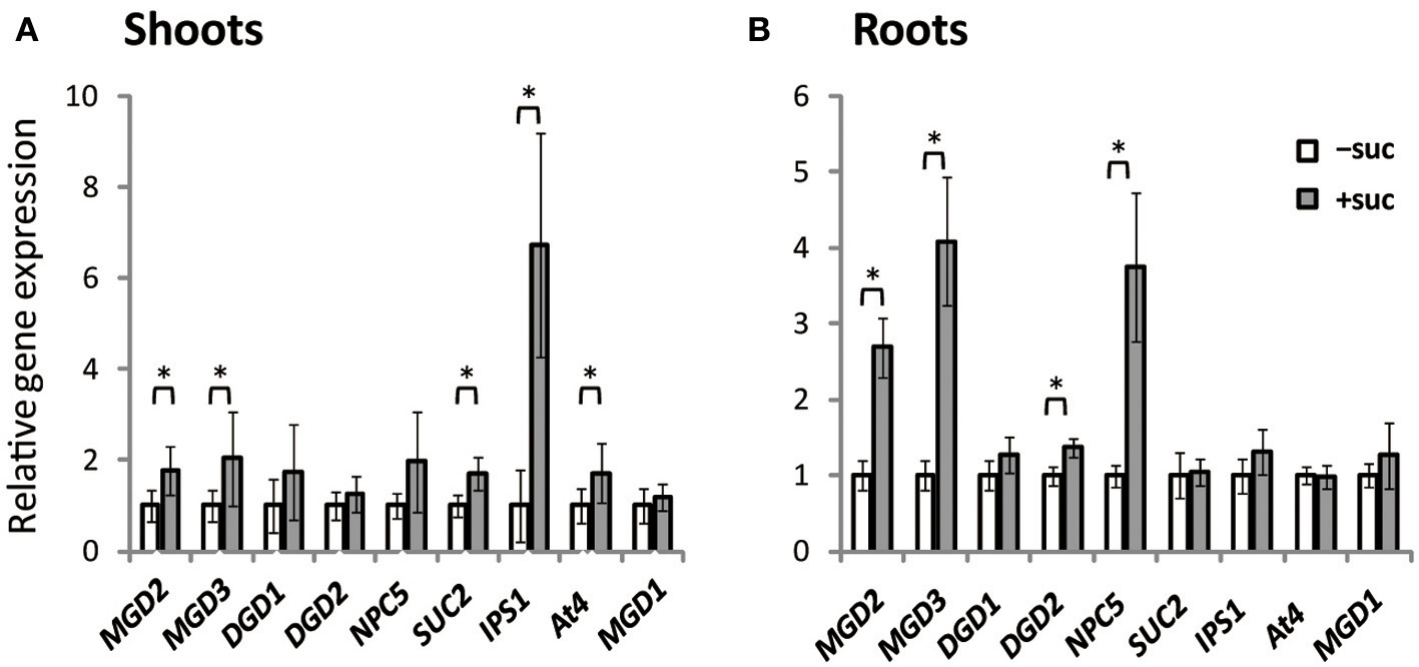
**Gene expression in *Arabidopsis* WT seedlings grown with or without exogenous sucrose**. Plants grown on MS agar with 1% (w/v) sucrose (+suc) or without sucrose [0.53% (w/v) mannitol as the osmotic control; −suc] for 7 d were then transferred to ½MS agar with or without sucrose, respectively, for 7 d. Relative mRNA abundance of genes upregulated during Pi starvation (*MGD2*, *MGD3*, *DGD1*, *DGD2*, *NPC5*, *IPS1*, and *At4*), a sucrose transporter gene (*SUC2*), and a Pi-non responsive galactolipid synthase gene (*MGD1*) in shoots **(A)** and roots **(B)** was analyzed by quantitative RT-PCR. Relative expression was normalized to the mRNA abundance of each respective gene under conditions without sucrose (−suc). Values represent the mean ± SD from three independent measurements. ^*^P < 0.05.

Expression of *MGD1*, *MGD2*, *MGD3*, *DGD1*, *DGD2*, *NPC5*, *SUC2*, *IPS1*, and *At4* was assessed using *Arabidopsis* plants grown on Murashige and Skoog (MS) agar supplemented with sucrose or the same molar concentration of mannitol as the osmotic control (Figure 1). *SUC2* expression in shoots was higher when sucrose was supplied (Figure 1A). Sucrose supplementation also increased the expression of type-B MGDG synthase genes, *MGD2* and *MGD3*, by ~2-fold compared with expression in the absence of sucrose. *NPC5* encodes non-specific phospholipase C5, which hydrolyzes PC (Gaude et al., 2008), and *DGD1* and *DGD2* encode proteins that synthesize DGDG (Härtel et al., 2000; Kelly and Dörmann, 2002; Kelly et al., 2003). Although *NPC5*, *DGD1*, and *DGD2* are responsive to Pi starvation and are involved in membrane lipid remodeling under Pi depletion (Härtel et al., 2000; Kelly and Dörmann, 2002; Kelly et al., 2003; Gaude et al., 2008), their expression in shoots was not significantly affected by sucrose supplementation (Figure 1A). *IPS1* and *At4* are non-protein-coding genes that are strongly and specifically induced by Pi starvation but are not related to lipid synthesis (Martín et al., 2000; Rubio et al., 2001; Bari et al., 2006; Narise et al., 2010). Expression of both *IPS1* and *At4* in shoots was increased by sucrose supplementation.

In roots, expression profiles differed slightly from those in shoots (Figure 1B). Expression of *MGD2* and *MGD3* increased markedly in roots when sucrose was supplied. Moreover, expression of *DGD2* and *NPC5* increased when sucrose was supplied, whereas sucrose supplementation did not alter the expression of *SUC2*, *IPS1*, or *At4*. These results suggested that sucrose and Pi may regulate translation in a mutually exclusive manner and be organ specific.

### Sucrose supplementation retards growth of a *MGD3* knockout mutant compared with WT

**Figure 2:**
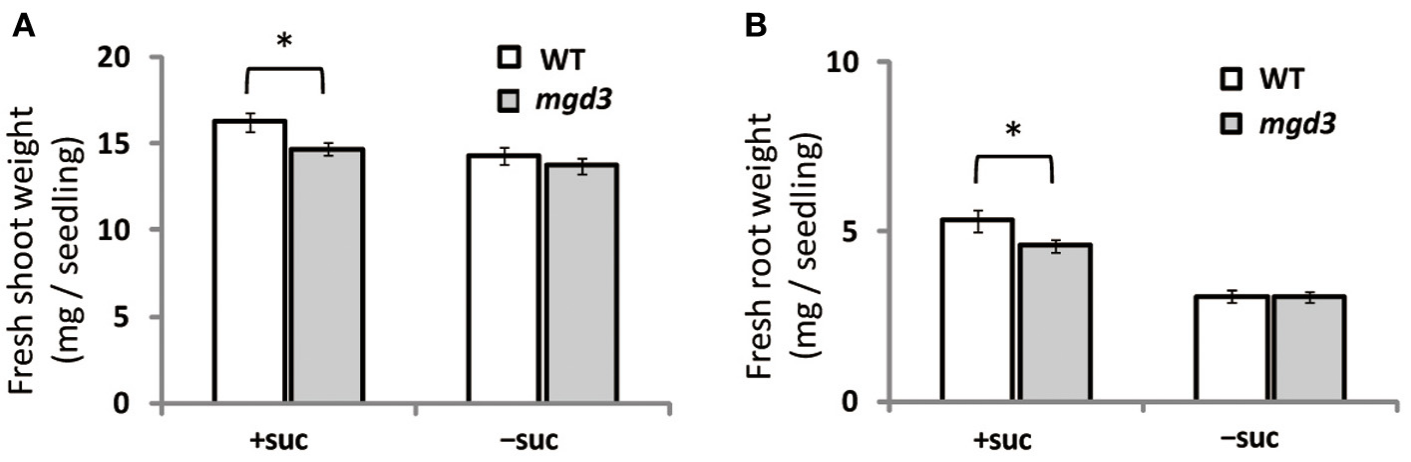
**Fresh weight of WT and the *mgd3* mutant under conditions with or without exogenously supplied sucrose**. Plants grown on MS medium supplemented with +suc or −suc for 7 d were then transferred to ½MS agar with or without sucrose, respectively, and grown for another 7 d. **(A)** Shoot fresh weight. **(B)** Root fresh weight. Values represent the mean ± SE from shoots and roots (*n* = 12 each). ^*^P < 0.05.

Supplementation of growth medium with sucrose promotes plant growth (Karthikeyan et al., 2007). To clarify whether type-B MGDG synthases are involved in the growth enhancement observed under sucrose supplementation, fresh weight of shoots and roots of a *MGD3* knockout mutant (*mgd3*) (Kobayashi et al., 2009a) was compared with that of WT (Figure 2). Without sucrose supplementation, the fresh weight of roots and shoots of *mgd3* was similar to that of WT (Figures 2A,B). When sucrose was supplied, however, fresh weight of shoot and root of *mgd3* was ~10 and ~14% lower compared with WT, respectively (Figures 2A,B). These data indicated that MGD3 plays a role in seedling growth under sucrose supplementation.

### Generation of transgenic *Arabidopsis* plants overexpressing *At*MGD3

**Figure 3:**
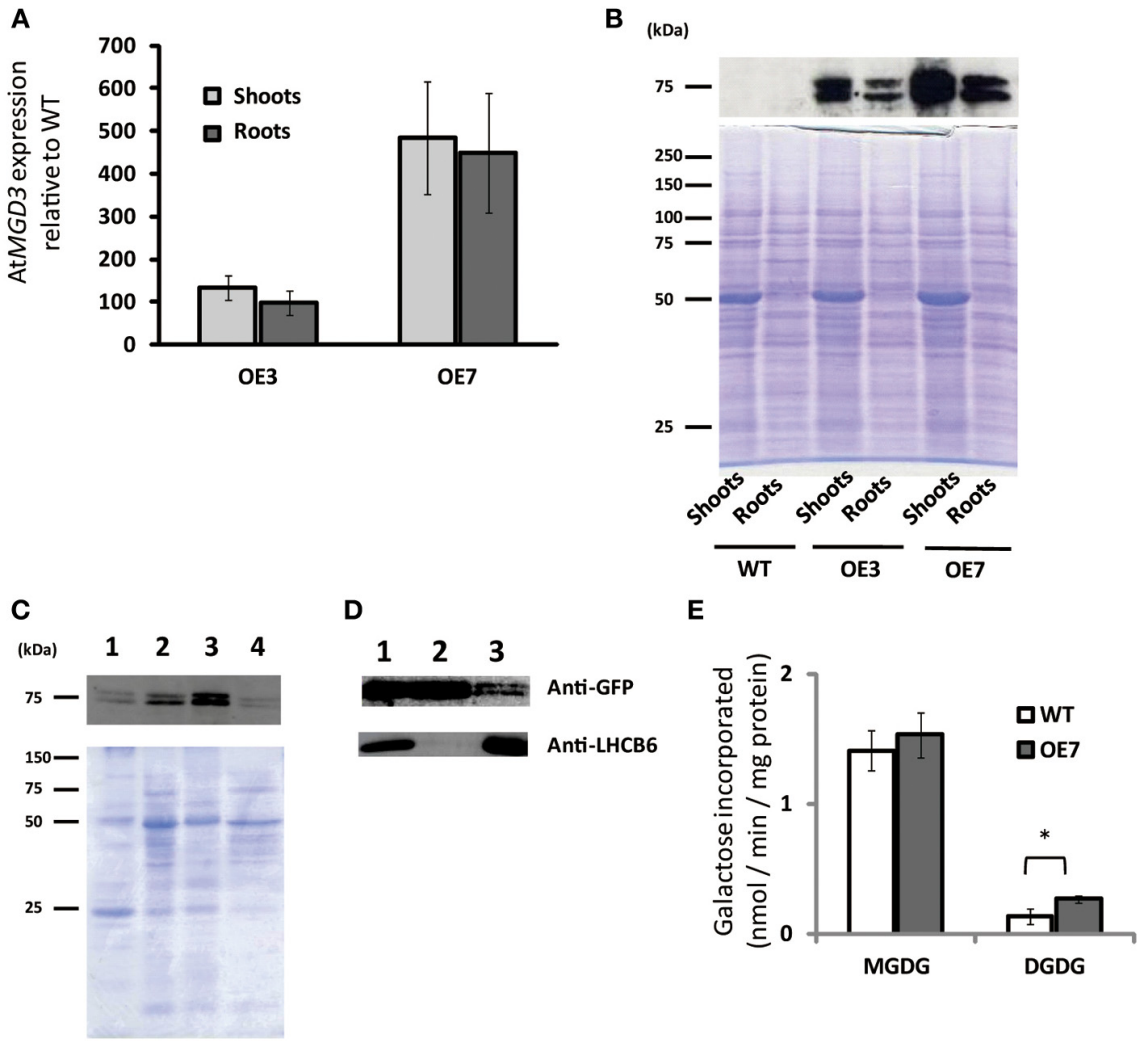
**Overexpression of *At*MGD3-GFP in *Arabidopsis***. Plants were grown on ½MS medium supplemented with 1% (w/v) sucrose (+suc) for 14 d. **(A)** Relative abundance of *AtMGD3* mRNA in shoots and roots of two overexpression lines (OE3 and OE7) was analyzed by quantitative RT-PCR. The relative expression was normalized to the abundance of *AtMGD3* mRNA in shoots and roots, respectively, of WT. Values represent the mean ± SD from three independent measurements. **(B)** Western blot analysis of *At*MGD3-GFP using anti-GFP. Crude-extract protein (20 μg from shoots, 10 μg from roots) was separated by SDS-PAGE, and bands were stained with Coomassie Brilliant Blue (lower panel) or transferred to a nitrocellulose membrane for western blotting (upper panel). **(C)** Microsomal localization of *At*MGD3-GFP. Crude extract from shoots of OE7 was fractionated into microsome and soluble fraction, and 10 μg proteins were separated by SDS-PAGE, and bands were stained with Coomassie Brilliant Blue (lower panel) or transferred to a nitrocellulose membrane for western blot analysis using anti-GFP (upper panel). **(D)** Chloroplast localization of *At*MGD3-GFP. Crude extract from shoots of OE7 (lane 1, 10 μg protein) was centrifuged at 2,000 ×g (supernatant, lane 2, 10 μg protein; pellet, lane 3, 30 μg protein). Anti–LHCB6 was used for a control. **(E)** Galactolipid synthetic activity. Microsormal fractions obtained from shoots of WT and OE7 were used for the assay. Values represent the mean ± SD from three independent measurements. ^*^P < 0.05.

To determine the role of MGD3 in plant growth under sucrose supplementation, we produced transgenic *Arabidopsis* plants that overexpressed *At*MGD3 fused to green fluorescent protein (GFP) and selected two overexpression (OE) lines (OE3 and OE7) for further analyses. In shoots, *MGD3* mRNA levels in OE3 and OE7 were ~130- and ~480-fold higher, respectively, than in WT (Figure 3A). In roots, *MGD3* mRNA levels in OE3 and OE7 were ~100- and ~450-fold higher, respectively, than in WT (Figure 3A).

Using antibodies against GFP, we also analyzed protein levels and subcellular localization in these transgenic plants (Figures 3B–D). In both OE3 and OE7, *At*MGD3-GFP was expressed in shoots and roots (Figure 3B). Moreover, the levels were higher in OE7 than in OE3. Thus, we used OE7 for further analyses. The subcellular localization of *At*MGD3-GFP in OE7 plants was analyzed after fractionation of the crude extract of shoots. In Figure 3C, crude extract was first centrifuged at low speed to obtain a thylakoid membrane–enriched fraction (Figure 3C, lane 1), and then the supernatant (Figure 3C, lane 2) was centrifuged at high speed to obtain two distinct fractions—the soluble fraction, which is enriched with soluble proteins (Figure 3C, lane 4), and the membrane fraction, which is enriched with microsomal membranes (Figure 3C, lane 3). The results showed that *At*MGD3-GFP mainly localized in microsomal membranes which is enriched with envelope membrane of chloroplasts and extraplastidial membranes (Figure 3C, lane 3). In Figure 3D, crude extract of OE7 (Figure 3D, lane 1) was centrifuged at low speed to obtain a chloroplast–enriched fraction (Figure 3D, lane 3) and the supernatant in which soluble proteins, chloroplast envelope membranes and extraplastidial membranes are major components (Figure 3D, lane 2). In Figure 3D, one of the light-harvesting chlorophyll a/b-binding (LHCB) proteins, LHCB6, was used as a marker protein of thylakoid membranes. Most of the *At*MGD3-GFP proteins localized in the envelope membrane-enriched fraction (Figure 3D, lane 2), but still small amount of *At*MGD3-GFP proteins was observed in the chloroplast-enriched fraction (Figure 3D, lane 3).

We also analyzed the galactolipid synthetic activity using the microsomal fractions obtained from WT and OE7 plants (Figure 3E). ^14^C-labeled UDP-galactose was used as a substrate for the galactolipid synthesis. As a result, microsomal fraction of OE7 plants showed higher levels of ^14^C-labeled DGDG compared with that of WT, whereas levels of ^14^C-labeled MGDG were not significantly different between WT and OE7 (Figure 3E). Regarding that MGDG produced by MGD3 on the outer envelope membrane of chloroplasts is sequentially supplied as a substrate to DGDG synthases, the result suggested that the localization of *At*MGD3-GFP is the outer envelope membrane as previously reported (Shimojima et al., 1997; Awai et al., 2001).

### Shoot fresh weight of overexpressing *At*MGD3 transgenic plants is higher than that of WT when sucrose is supplied

**Figure 4:**
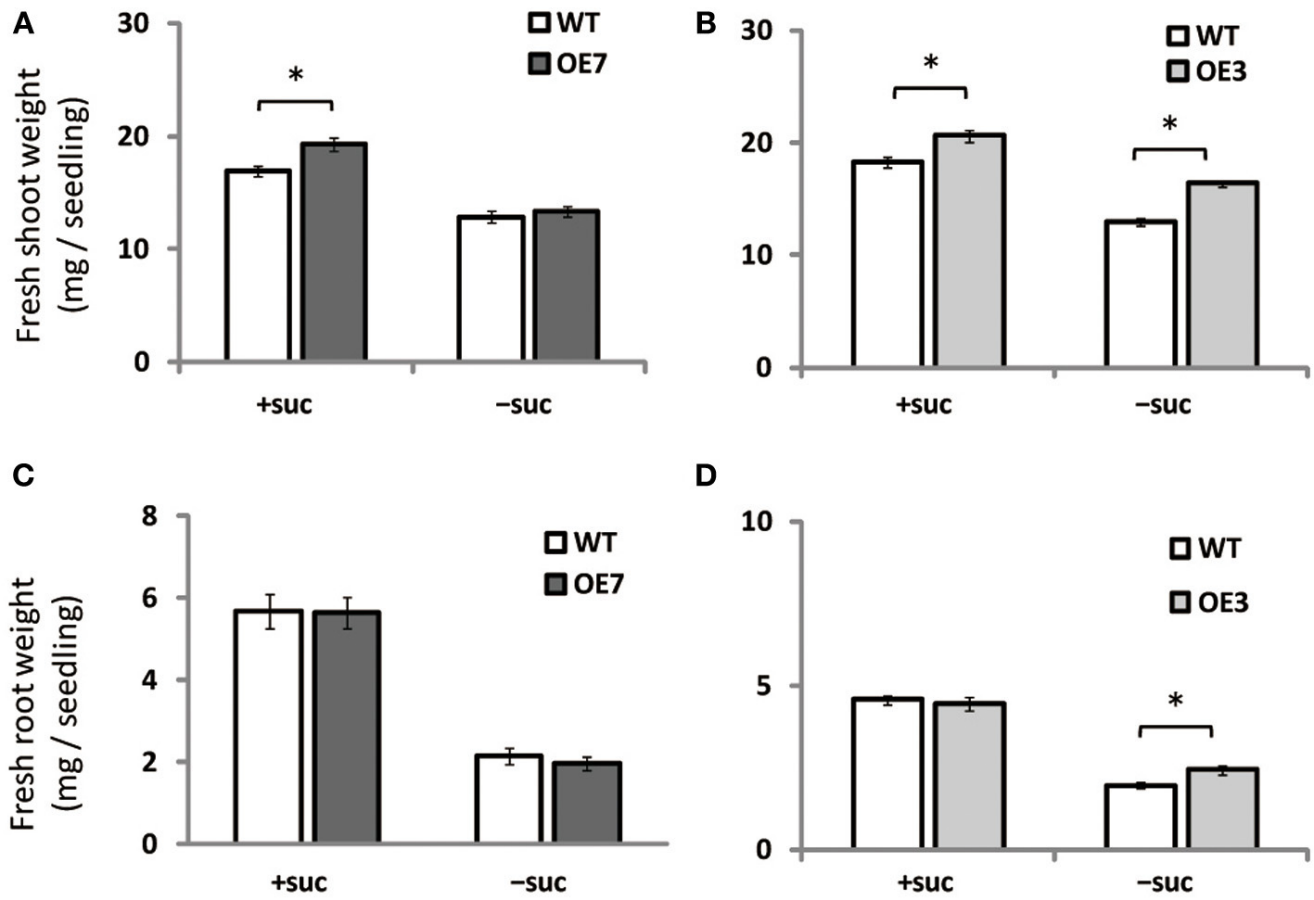
**Fresh weight of *Arabidopsis* WT and the OE7 and OE3 transformant overexpressing *At*MGD3-GFP in the absence or presence of exogenous sucrose**. Plants grown on MS medium supplemented with +suc or −suc for 7 d were then transferred to ½MS agar with or without sucrose, respectively, and grown for another 7 d. Shoot fresh weight of OE7 **(A)** and OE3 **(B)**. Root fresh weight of OE7 **(C)** and OE3 **(D)**. Values represent the mean ± SE (*n* = 12 groups of 3–5 plants for OE7 shoots, *n* = 6 groups of 3–5 plants for OE7 roots, and *n* = 20 groups of 3–5 plants for OE3 shoots and roots). ^*^P < 0.05.

When grown on medium supplemented with sucrose, shoot fresh weight of OE3 and OE7 was ~13 and ~14% higher than that of WT, respectively (Figures 4A,B). When grown on medium without sucrose, however, shoot fresh weight was comparable between WT and OE7 (Figures 4A,B), whereas OE3 showed ~26% higher shoot fresh weight compared with WT. Root fresh weight was similar between WT and OE7 regardless of sucrose availability (Figure 4C), whereas OE3 showed higher root fresh weight compared with WT when sucrose was not supplemented to the growth medium (Figure 4D). From these results, it was suggested that OE of MGD3 enhances plant growth regardless of sucrose supplementation to the growth medium.

### Relative amount of DGDG in membrane lipids is higher in OE7 than in WT in both shoots and roots

**Figure 5:**
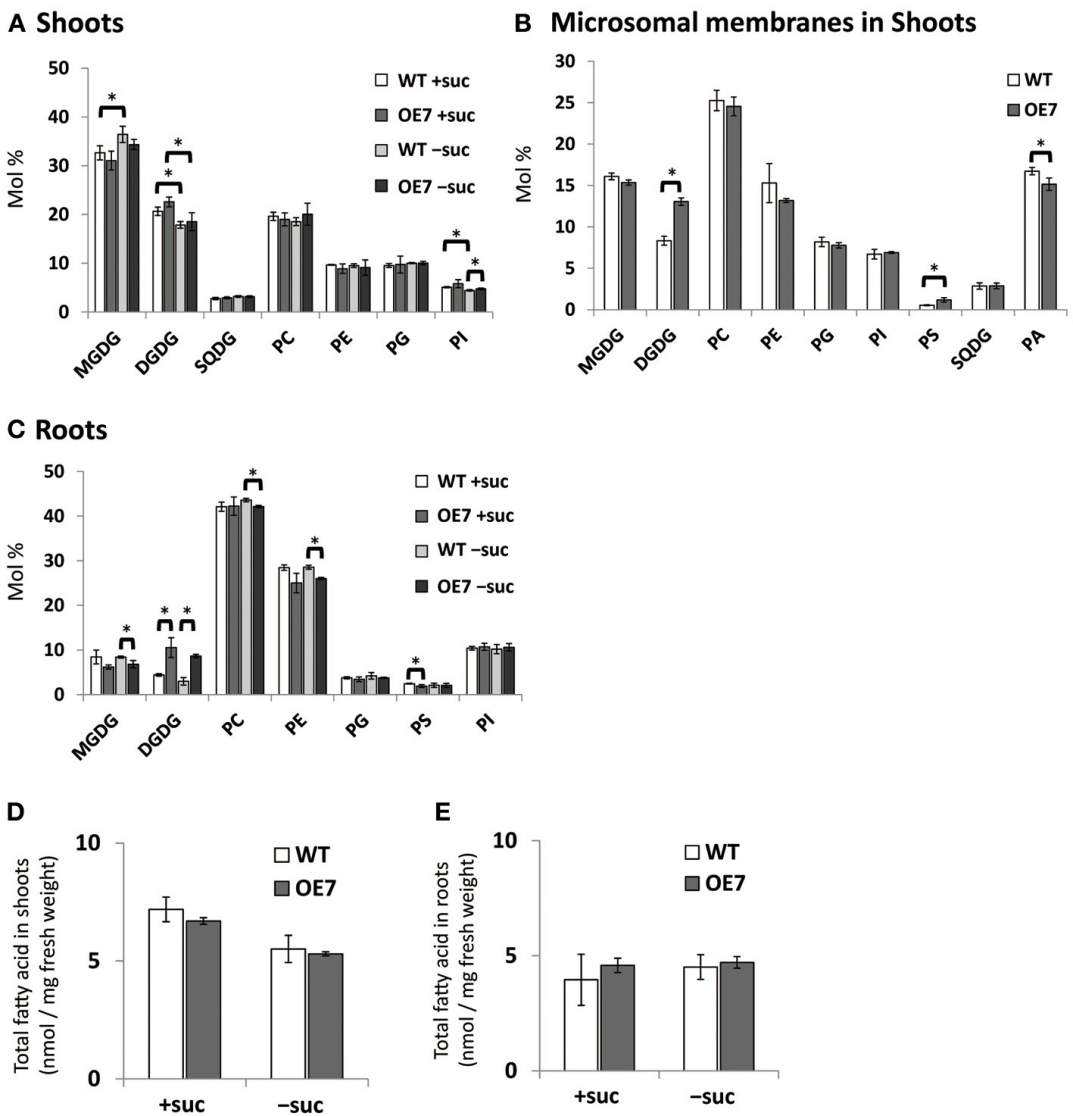
**Lipid analysis of WT and OE7 seedlings grown with or without exogenous sucrose**. Molar ratio of membrane lipids of shoots **(A)** and roots **(C)**, and of microsomal lipids of shoots **(B)**, and total fatty acid content in shoots **(D)** and roots **(E)**. Plants were grown on MS medium supplemented with +suc or −suc for 10 d **(A,C)** or 7 d **(B,D,E)** were then transferred to ½MS agar with or without sucrose, respectively, and grown for another 10 d **(A,C)** or 7 d **(B,D,E)**. MGDG, monogalactosyldiacylglycerol; DGDG, digalactosyldiacylglycerol; PC, phosphatidylcholine; PE, phosphatidylethanolamine; PG, phosphatidylglycerol; PI, phosphatidylinositol; PS, phosphatidylserine; SQDG, sulfoquinovosyldiacylglycerol; PA, phosphatidic acid. Values represent the mean ± SD from three independent measurements. ^*^P < 0.05.

Although the effect of Pi starvation on membrane lipid composition has been well studied, the composition under sucrose supplementation has not been assessed. Thus, we assessed membrane lipid composition in shoots and roots of WT and OE7 plants grown on medium containing sucrose (Figure 5A). In shoots of WT plants, the molar ratio of DGDG in the total membrane lipids was increased when sucrose was supplemented, whereas that of MGDG was decreased (Figure 5A). Although the level of *MGD3* mRNA in OE7 under sucrose supplementation was markedly higher than that observed in WT plants grown under Pi depletion (Figure 3A; Narise et al., 2010), only a small increase in DGDG mol% was observed in OE7 shoots compared with WT shoots (Figure 5A). Thus, we also analyzed lipid composition of the microsomal fraction extracted from shoots of WT and OE7 supplemented with sucrose (Figure 5B). Microsomal fraction was obtained using the same method as described in Figure 3C. In WT plants grown under normal growth conditions, MGDG mainly localizes in the thylakoid membrane and the molar ratio of MGDG and DGDG is 2:1 as shown in Figure 5A. In the microsomal fraction, ratio of MGDG in the total lipids (~16 mol%) were comparable between WT and OE7 (Figure 5B), indicating that the amounts of thylakoid membrane included in the fractions were similar between WT and OE7. However, microsomal fraction of OE7 contained higher amount of DGDG (~13 mol%) than that of WT (~8 mol%, Figure 5B). From these results, it was suggested that DGDG increased in OE7 plants was translocated to extraplastidial membranes. The increase in DGDG mol% in roots was more significant than that in shoots when compared OE7 with WT (Figure 5C). When sucrose was supplied to WT plants, transcript levels of *NPC5* and *DGD2* were significantly increased in roots but not in shoots (Figures 1A,B). These results together suggested that the marked increase in DGDG mol% in plants requires a simultaneous increase in transcript levels of not only *MGD2/MGD*3 but also *NPC5* and *DGD2*. Regarding the membrane lipid composition under Pi depletion that we used in this experiment, it is known that DGDG mol% increases whereas PC and phosphatidylethanolamine (PE) molar ratios decrease (Kobayashi et al., 2009a). Under sucrose supplementation, DGDG mol% increased, but a decrease in molar ratios of PC and PE was not observed in either shoots or roots (Figures 5A,C). We also analyzed the amount of total fatty acids, and confirmed that there was no difference of the amount of membrane lipids between WT and OE7 supplemented with or without sucrose (Figure 5D,E).

### Free inorganic Pi content is comparable between WT and OE7 plants

**Figure 6:**
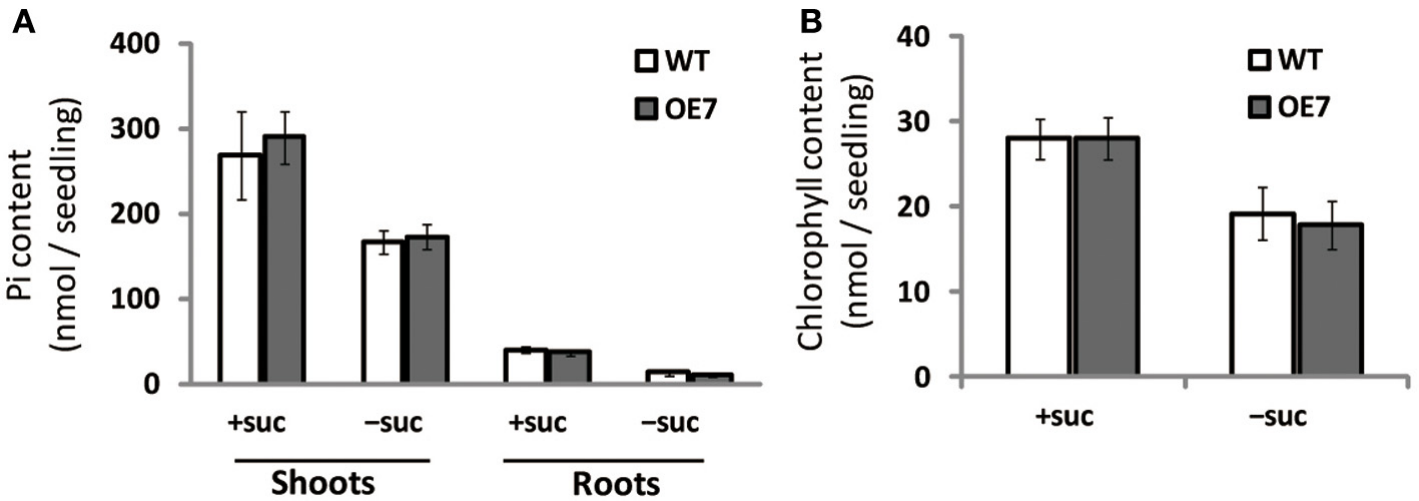
**Pi (A) and chlorophyll (B) content in WT and OE7 seedlings grown with or without exogenous sucrose**. Plants grown on MS medium supplemented with +suc or −suc for 7 d were then transferred to ½MS agar with or without sucrose, respectively, and grown for another 7 d. Values represent the mean ± SD from three independent measurements.

Under Pi depletion, plant cells utilize phosphorus by degrading phospholipids in biological membranes, and DGDG compensates for loss of phospholipids in the membranes; thus, increased amounts of available Pi in cells are utilized for other essential biological processes. To test if the increase in DGDG mol% might affect the levels of available Pi in cells without changing phospholipid levels, we measured free inorganic Pi content in WT and OE7 plants (Figure 6A). In both shoots and roots, WT and OE7 contained the same amount of Pi, clearly showing that the increase in DGDG mol% in the membrane does not affect the concentration of free Pi in the cells.

### Chlorophyll content and photosynthetic activity are similar between WT and OE7 plants

Regardless of sucrose supplementation, chlorophyll content per seedling did not differ significantly between WT and OE7 (Figure 6B). We also measured the photosynthetic activity (relative to chlorophyll content) in WT and OE7 plants grown on sucrose-supplemented medium (Table 1). No significant difference was observed between WT and OE7, suggesting that the enhanced growth of OE7 under sucrose supplementation was not due to higher photosynthetic activity compared with WT.

**Table 1:** **Chlorophyll fluorescence parameters for WT and OE7**.

	**WT**	**OE7**
*Fv*/*Fm*	0.83 ± 0.00	0.82 ± 0.04
qP	0.83 ± 0.01	0.88 ± 0.04
NPQ	0.23 ± 0.06	0.32 ± 0.02
ϕII	0.55 ± 0.02	0.60 ± 0.08

*Plants were grown on ½MS agar supplemented with 1% (w/v) sucrose for 2 weeks. Chlorophyll fluorescence parameters represent the mean ± SD of three replicates. qP, photochemical quenching; NPQ, non-photochemical quenching*.

### Sucrose content in shoots is lower in OE7 than in WT under sucrose supplementation

**Figure 7:**
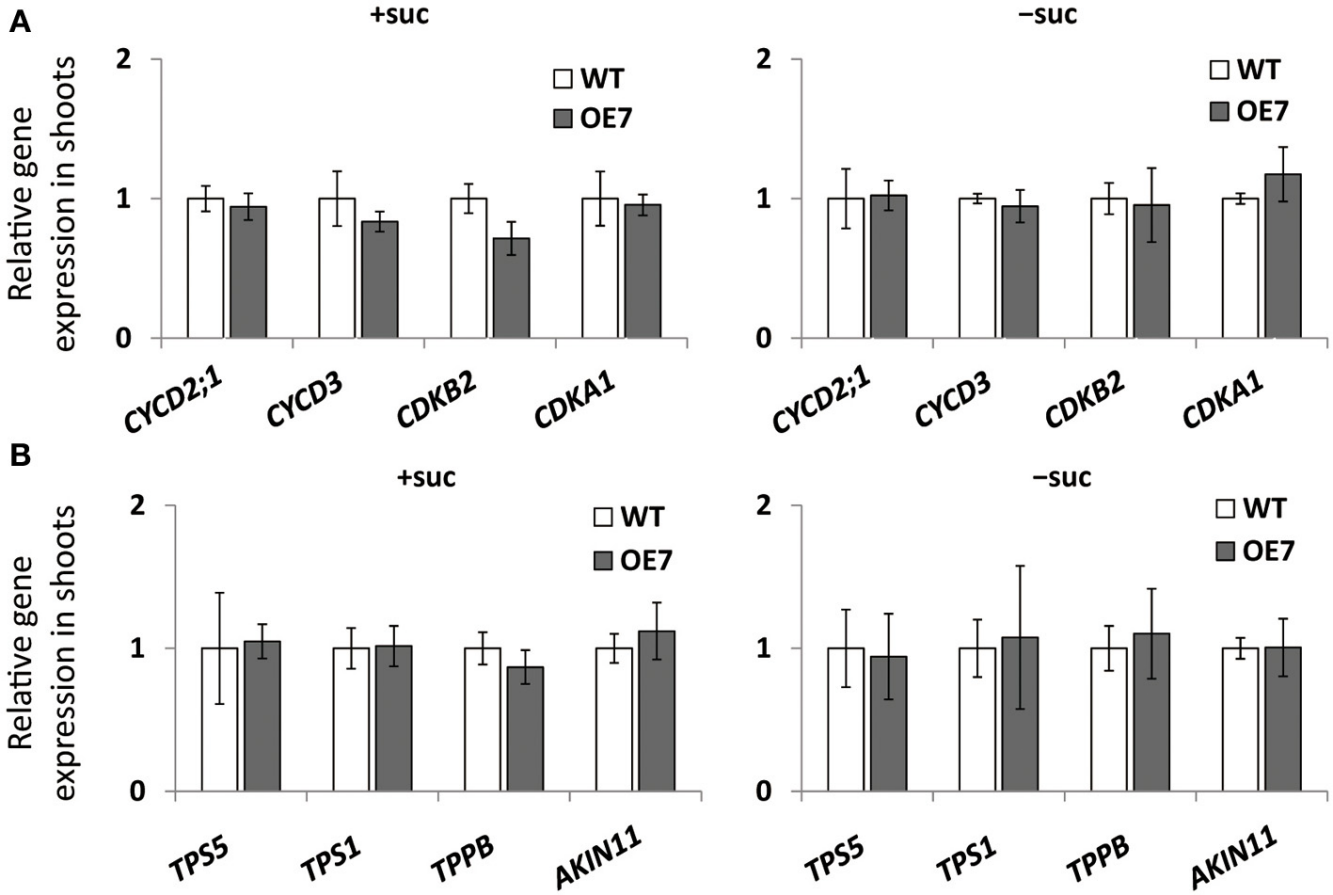
**Cell cycle–related gene (A) and trehalose-6-phosphate (T6P)-metabolism-related gene (B) expression in WT and OE7 seedlings**. Plants grown on MS agar with +suc or −suc for 7 d were then were transferred to ½MS agar with or without sucrose, respectively, for another 7 d. Relative mRNA abundance of cell cycle–related genes (*CYCD2;1*, *CYCD3*, *CDKB2*, *CDKA1*) and of T6P-metabolism-related genes (*TPS5*, *TPS1*, *TPPB*, *ALIN11*) in shoots were analyzed by quantitative RT-PCR. Relative expression was normalized to the corresponding mRNA abundance in WT. Values represent the mean ± SD from three independent measurements.

**Figure 8:**
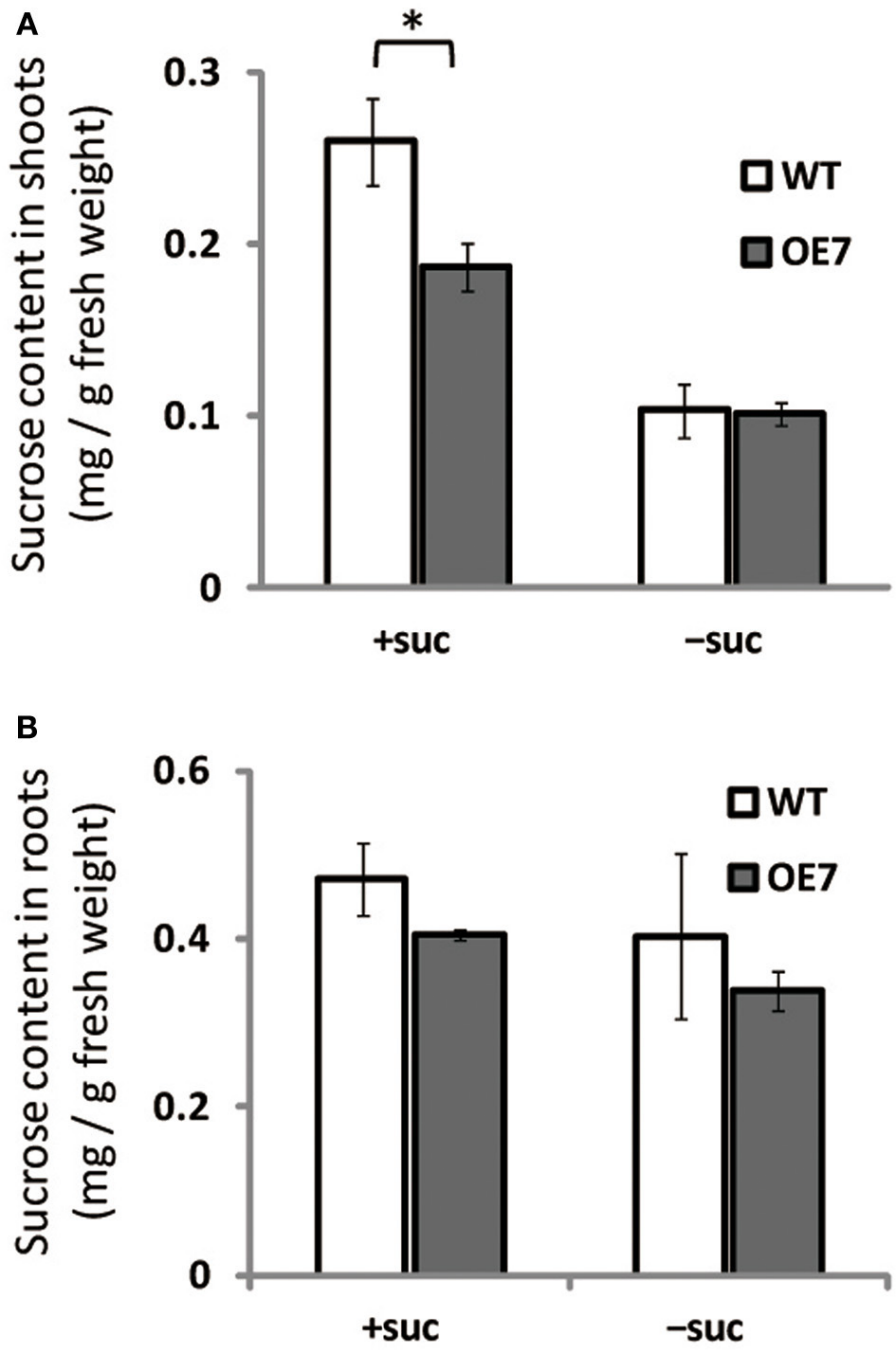
**Sucrose content in WT and OE7 seedlings grown with or without exogenous sucrose**. Plants grown on MS medium supplemented with +suc or −suc for 7 d were then transferred to ½MS agar with or without sucrose, respectively, and grown for another 7 d. **(A)** Sucrose content in shoots. **(B)** Sucrose content in roots. Values represent the mean ± SE [*n* = 6 (shoot, +suc), *n* = 5 (shoot, −suc), *n* = 3 (root, +suc and −suc)]. ^*^P < 0.05.

We observed enhanced growth of OE7 plants only when sucrose was supplied in the growth medium (Figure 4A). Sucrose is a major mobile form of photoassimilates, but there were no significant differences in photosynthetic activity between WT and OE7 (Table 1). Thus, we also analyzed expression levels of genes involved in cell cycle and trehalose-6-phposphate metabolism (Figure 7). Trehalose-6-phosphate metabolism and its content in plants are known to be related to growth enhancement under sucrose-supplemented conditions (Zhang et al., 2009; Debast et al., 2011; Delatte et al., 2011; Martínez-Barajas et al., 2011). However, expression levels of genes were comparable between WT and OE7 (Figures 7A,B). Thus, to clarify whether the growth difference was due to the uptake efficiency of the exogenously supplied sucrose, we first measured the sucrose concentration in shoots and roots of WT and OE7 in the absence or presence of sucrose (Figure 8). In both shoots and roots, the sucrose concentration in plants grown without exogenous sucrose was comparable between WT and OE7 (Figure 8). When sucrose was supplied, its concentration—especially in shoots—of WT and OE7 was higher than that in plants grown without sucrose (Figure 8A). In shoots of OE7 and WT grown with sucrose, sucrose concentration was 1.7- and 2.2-fold higher, respectively, compared with OE7 and WT grown without sucrose (Figure 8A). As a result, the sucrose concentration in shoots of OE7 was ~26% lower than that of WT only under sucrose supplementation. Sucrose supplementation did not significantly affect the sucrose content in roots (Figure 8B).

### Galactolipid synthesis using exogenously supplied sucrose is enhanced in OE7 plants

**Figure 9:**
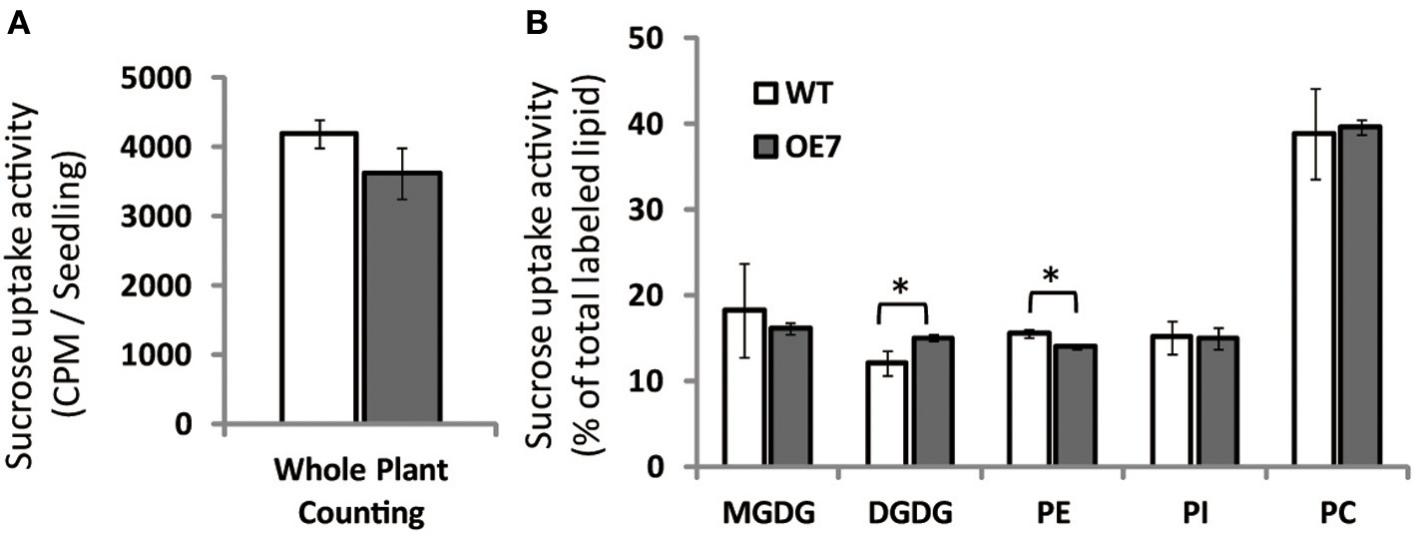
**Sucrose uptake of WT and OE7 plants**. Plants were grown on sucrose-free ½MS for 2 weeks and then transferred to ^14^C-labeled sucrose containing ½MS for 4 h. **(A)** Counts of ^14^C-labeled whole seedlings. **(B)** Relative intensity of ^14^C-labeled membrane lipids. Total lipid extracted from shoots and roots after ^14^C labeling was separated by thin-layer chromatography, and intensities of each ^14^C-labeled lipid were analyzed. Values represent the mean ± SD from three independent measurements. ^*^P < 0.05.

To clarify the reason for the observed decrease in sucrose concentration in shoots of OE7 plants under sucrose supplementation, we measured the uptake of [^14^C]sucrose. Unexpectedly, the levels of labeled seedlings did not differ significantly between WT and OE7, suggesting that the efficiency of sucrose uptake did not differ between WT and OE7 (Figure 9A). However, the relative amount of ^14^C incorporation in each membrane lipid clearly indicated that sucrose absorbed from roots was immediately utilized as a carbon source for membrane lipid synthesis, and the ratio of ^14^C level in DGDG relative to all labeled lipids in OE7 was slightly higher than that in WT (Figure 9B). These results suggested that sucrose uptake activity of OE7 is similar to that of WT, but the galactolipid biosynthesis using exogenously supplied sucrose as a carbon source was enhanced in OE7 compared with WT.

## Discussion

When sucrose was exogenously supplied to the growth medium, the expression of *MGD2* and *MGD3* as well as *SUC2*, *IPS1*, and *At4* was upregulated in shoots (Figure 1A). However, expression levels of three genes responsive to Pi deficiency also involved in lipid synthesis (*DGD1*, *DGD2*, and *NPC5*) were not significantly changed in shoots (Figure 1A). Our result agrees with previous studies reporting that the change in gene expression induced by sucrose supplementation is similar to that induced by Pi deficiency (Hammond et al., 2003; Vance et al., 2003; Wu et al., 2003; Misson et al., 2005; Müller et al., 2005, 2007; Hammond and White, 2008). However, this similarity is not a common feature of all membrane-remodeling genes responsive to Pi deficiency. The *Arabidopsis hsp1* mutant, in which sucrose content is higher than in WT in both shoots and roots, shows a hypersensitive phenotype in response to Pi starvation, suggesting that sucrose is a global regulator of plant responses to Pi starvation (Lei et al., 2011). Indeed, microarray analysis of the *hsp1* mutant has revealed the induction of ~70% of the Pi starvation–responsive genes in WT (Lei et al., 2011). *MGD3* is one of the top 20 genes that are synergistically induced by Pi starvation in the *hsp1* mutant, although expression of *DGD1* and *DGD2* is comparable between WT and *hsp1* and *NPC5* is 5-fold lower in *hsp1* than in WT (Lei et al., 2011). Under Pi depletion, a simultaneous increase in expression levels of these genes occurs in shoots, which is essential for membrane lipid remodeling (Benning and Ohta, 2005; Nakamura, 2013). Thus, although the time course of gene expression should be further analyzed, the responses to exogenously supplied sucrose and Pi starvation do not appear to be fully correlated.

Growth of transgenic plants that overexpress *MGD3* was enhanced compared with WT, whereas growth of the *MGD3* knockout mutant *mgd3* was slower than WT under sucrose supplementation (Figures 2, 4A,B). Unexpectedly, although *MGD3* expression was significantly higher in OE7 than in WT (Figure 3A), the DGDG molar ratio in membrane lipids was only slightly different between OE7 and WT (Figure 5). Indeed, the level of *MGD3* mRNA is markedly higher in OE7 than in WT plants grown under Pi depletion (Narise et al., 2010). In shoots of WT grown under Pi depletion, the molar ratio of DGDG in the total membrane lipids is ~15% higher than in plants grown under Pi-sufficient conditions (Kobayashi et al., 2009a). The effect of *MGD3* OE on membrane lipid composition may have been smaller than we expected because of the lack of co-activation of genes/enzymes involved in DGDG synthesis. When DGDG synthesis is not activated, MGDG may accumulate in the membrane. However, MGDG is not a bilayer-forming lipid in the membrane, and thus MGDG hyperaccumulation might be cytotoxic (Murphy, 1986). Indeed, rough cell membrane surfaces and defects in cell division are observed in *Escherichia coli* that accumulate MGDG (Gad et al., 2001). Our results suggest that a feedback mechanism or other unknown mechanisms might exist to prevent hyperaccumulation of MGDG in membranes.

Inorganic Pi content in plant cells (Figure 6A), photosynthesis (Table 1), and cell division–related gene expression (Figure 7A) were not significantly different between WT and OE7 plants. We also measured the sucrose content in shoots and found that OE7 contained less sucrose than WT (Figure 8). Given that sucrose uptake by OE7 and WT plants was similar (Figure 9), upregulation of galactolipid synthesis mediated by type-B MGDG synthases in the outer envelope membrane of chloroplasts may correlate with accelerated sugar utilization as a carbon source only when the carbon source is supplied exogenously.

When carbon availability is elevated by sucrose supplementation, trehalose 6-phosphate (T6P) content increases (Schluepmann et al., 2004; Lunn et al., 2006). T6P inhibits the catalytic activity of the SNF1-related protein kinase SnRK1 in growing tissues of plants (Zhang et al., 2009; Debast et al., 2011; Delatte et al., 2011; Martínez-Barajas et al., 2011). Inhibition of SnRK1 blocks expression of more than 1000 genes involved in biosynthesis, growth, and stress responses (Baena-González et al., 2007; Nunes et al., 2013). *MGD3* is not included among the genes regulated by SnRK1, suggesting that its transcriptional regulation by exogenously supplied sucrose is distinct from the SnRK1–related regulation mechanism. Moreover, expression levels of several genes involved in T6P metabolism and the SnRK1-mediated signaling pathway were comparable between WT and OE7 (Figure 7B), showing that there was no correlation between upregulation of MGDG synthesis and SnRK1-mediated stress responses. Thus, upregulation of MGD3 appears to be involved in sucrose metabolism and growth enhancement under sucrose supplementation in a manner different from that mediated by T6P and SnRK1.

Recently, it was suggested that balance between available Pi and carbon content might be important for the response to Pi starvation (Lei and Liu, 2011). Given that *MGD3* expression levels were increased by Pi starvation and sucrose supplementation (Figure 1) and that sucrose content was lower in shoot tissues of OE7 than of WT when sucrose was supplied (Figure 8A), galactolipid synthesis on the outer envelope membrane of chloroplasts might play the following two roles: (1) maintenance of the ratio of available Pi and carbon in plant tissues by reducing the cellular sucrose content via galactolipid synthesis in Pi-depleted growth medium, (2) supply of DGDG as a component of the plasma membrane to support enhanced growth under sucrose supplementation. Regardless of which scenario is correct, future work will be needed to confirm a new role for MGD3 other than for galactolipid supply during lipid remodeling under Pi depletion.

## Materials and methods

### Plant material and growth conditions

Seedlings of WT *A. thaliana* (Columbia-0), *mgd3* mutant and transformants overexpressing *At*MGD3-GFP protein were grown on Murashige and Skoog (MS) medium (Murashige and Skoog, 1962) solidified with 0.8% (w/v) agar containing 1% (w/v) sucrose or 0.53% (w/v) mannitol, for the osmotic control, at 23°C under continuous white light.

MGD3 OE transformants were produced by a modified version of the vacuum-infiltration method (Bechtold and Pelletier, 1998) using pBI121 in which the *AtMGD3* cDNA sequence and *GFP* tag were inserted under control of the 35S-CaMV promoter, and transformants were then selected on MS agar containing 50 μg·mL^−1^ kanamycin.

### Quantitative RT-PCR

Total RNA was extracted from plant shoots and roots using the SV Total RNA Isolation System (Promega). Reverse transcription (RT) was performed using the PrimeScript RT reagent kit (Takara). PCR was conducted using the SYBR Premix Ex Taq II (Takara), and signals were detected/quantified using the Thermal Cycler Dice Real Time System (Takara). Quantitative RT-PCR was carried out as following method. Each PCR reaction mixture (25 μL) was prepared to contain cDNA (RT product from 6 ng of RNA), 10 μL SYBR Premix Ex Taq II and 0.4 μM of each primer. Samples were run for 40 cycles under the following thermal cycling protocol and analyzed the dissociation curve: preheating step at 95°C for 30 s, 40 amplification cycles of 95°C for 5 s, 60°C for 30 s, 1 cycle of 95°C for 15 s, 60°C for 30 s, 95°C 15 s. Quantitative RT-PCR was carried out using *AtUBQ10* (At4g05320) as an internal standard. The following primers were used:

AtMGD3_Fw: 5′ TCGTGGCGGATTGGTTTAG 3′
AtMGD3_Rv: 5′ CGTTGTTGTTGTTGGGATAGATG 3′
AtMGD2_Fw: 5′ GATTCGATCACTTCCTATCATCCTC 3′
AtMGD2_Rv: 5′ TGTGCTAAACCATTCCCCAAC 3′
AtDGD1_Fw: 5′ CTGAAGAGAGATCCCGTGGTG 3′
AtDGD1_Rv: 5′ TCCCAAGTTCGCTTTTGTGTT 3′
AtDGD2_Fw: 5′ TGCAGAACCTATGACGATGGA 3′
AtDGD2_Rv: 5′ GCTCTGTAAGTTGCGATGGTTG 3′
AtNPC5_Fw: 5′ TTCTTCATCTCCCCTTGGATTG 3′
AtNPC5_Rv: 5′ GTGACATTAGGTACGGCCCATT 3′
AtSUC2_Fw: 5′ TCCCTTTCCTTCTCTTCGACAC 3′
AtSUC2_Rv: 5′ CATAAGCCCCAAAGCACCA 3′
AtIPS1_Fw: 5′ AGACTGCAGAAGGCTGATTCAGA 3′
AtIPS1_Rv: 5′ TTGCCCAATTTCTAGAGGGAGA 3′
At4_Fw: 5′ CTGAAGCTCAAGAACCCTCTGAA 3′
At4_Rv: 5′ CCTCTCAAAACCCTTTATTGGTGA 3′
AtMGD1_Fw: 5′ AGGTTTCACTGCGATAAAGTGGTT 3′
AtMGD1_Rv: 5′ AACGGCAATCCCTCCTCAC 3′
AtCYCD2;1_Fw: 5′ GCTGCTGCAGTGTCTGTTTC 3′
AtCYCD2;1_Rv: 5′ ACAGCTCTTACCGCAACTCG 3′
AtCYCD3_Fw: 5′ CAACTACCAGTGGACCGCATC 3′
AtCYCD3_Rv: 5′ AATCACGCAGCTTGGACTGTT 3′
AtCDKB2_Fw: 5′ CCAATGAAGAAGTATACCCATGAGA 3′
AtCDKB2_Rv: 5′ AATGGGTGGCACCAAGAAG 3′
AtCDKA1_Fw: 5′ CCGAGCACCAGAGATACTCC 3′
AtCDKA1_Rv: 5′ GTTACCCCACGCCATGTATC 3′
AtTPS5_Fw: 5′ TCTCGGTTTGGGTGCAGAGCA 3′
AtTPS5_Rv: 5′ ACCAAACTCGACGTTTCCCAGTCT 3′
AtTPS1_Fw: 5′ ACCATAGTTGTTCTGAGCGGAAGCA 3′
AtTPS1_Rv: 5′ TCATCCACTCTCCATTCGTAAGCCT 3′
AtTPPB_Fw: 5′ GGGACAAGGGCCAGGCACTC 3′
AtTPPB_Rv: 5′ ACACCGGCACAACATCATCCGA 3′
AtAKIN11_Fw: 5′ CACCATTCCTGAGATCCGTCA 3′
AtAKIN11_Rv: 5′ GAGACAGCAAGATAACGAGGGAG 3′
AtUBQ10_Fw: 5′ CCCTAACGGGAAAGACGATTAC 3′
AtUBQ10_Rv: 5′ AAGAGTTCTGCCATCCTCCAAC 3′

Sequences of AtTPS5_Fw and Rv, AtTPS1_Fw and Rv, AtTPPB_Fw and Rv, and AtAKIN11_Fw and Rv were described by Nunes et al. (2013). Sequences of AtMGD2_Fw and Rv, AtIPS1_Fw and Rv, and At4_Fw and Rv were described by Narise et al. (2010). Sequences of AtCYCD2;1_Fw and Rv, CDKA1_Fw and Rv were described by Sanz et al. (2011).

### Fresh weight measurement

Fresh weight of shoots or roots from 3 to 5 plants were measured together, and the average weight of an individual plant was calculated. The mean ± SE was calculated furthermore.

### Western blot analysis

For western blotting, WT and OE plant samples were homogenized in 50 mM Tris-HCl, pH 7.5 and centrifuged at 3,000 ×g to remove tissue debris, and each supernatant was used as crude extract. Of the crude protein (20 μg from shoots, 10 μg from roots) was subjected to SDS-PAGE (12.5% polyacrylamide), blotted onto a nitrocellulose membrane (Whatman), and incubated for 3 h at 23°C with monoclonal anti-GFP (Clontech**;** diluted 1:5,000) and then with horseradish peroxidase–conjugated anti-mouse IgG secondary antibody (Thermo Scientific; diluted 1:100). Bands were detected by chemiluminescence substrates (SuperSignal West Femto Chemiluminescent Substrate, Thermo Scientific) and film (Hyperfilm ECL, GE Healthcare).

To further assess subcellular localization, crude-extract proteins were centrifuged at 125,000 × g to yield soluble and microsomal membrane fractions. These fractions were then subjected to SDS-PAGE/western blotting as described above, and protein bands were detected using Image Quant LAS 500 (GE Healthcare).

For chloroplast purification, plants were homogized by a blender in lysis buffer (50 mM HEPES-KOH, 330 mM sorbitol, 2.0 mM EDTA, 1.0 mM MgCl_2_, 1.0 mM MnCl_2_, pH7.8) with Protease Inhibitor Cocktail (Roche; complete Mini), and centrifuged at 2,000 × g for 5 min to enrich chloroplasts as the pellet. Resuspended pellet and supernatant fractions were then subjected to SDS-PAGE/western blotting as described above, or for the LHCB6 detection, membranes were incubated for 3 h at 23°C with monoclonal anti-LHCB6 (Agrisera**;** diluted 1:5,000) and then with peroxidase anti-rabbit IgG secondary antibody (Vector Laboratories; diluted 1:10,000). Protein bands were detected using Image Quant LAS 500 (GE Healthcare).

### Measurement of galactolipid synthase activity

Plant microsomal fractions of WT and OE7 were obtained by centrifugation of homogenized plant shoots at 3,000 × g (supernatant) and 125,000 × g (pellet). Galactolipid synthetic activities were measured using ^14^C-labeled UDP-galactose as a substrate according to our previous reports with minor modifications (Yamaryo et al., 2003; Shimojima et al., 2013). Briefly, after pre-incubation of microsomal enzyme in 190 μL of assay mixture [6.4 mM dioleoylglycerol in 0.01% (w/v) Tween 20, 10 mM dithiothreitol, 10 mM sodium acetate, and 18 mM MOPS-KOH, pH 7.8] at 30°C for 5 min, 10 μL of ^14^C-labeled UDP-galactose (8.08 mM, 91.6 Bq nmol^−1^) was added to start the reaction. The reaction products were extracted in ethyl acetate, separated by thin layer chromatography (solvent system, acetone: toluene: water = 136: 45: 13, v/v/v), and quantified using a fluoro-image analyzer (FLA-7000, Fujifilm).

### Lipid analysis

Total lipid was extracted according to Bligh and Dyer (1959). The polar membrane lipids were separated by two-dimensional silica gel thin-layer chromatography (Kobayashi et al., 2007). Separated lipids were then subjected to hydrolysis and methylation, and fatty acid methyl esters were quantified by gas chromatography using pentadecanoic acid as an internal standard (Kobayashi et al., 2006). Microsomal membranes were obtained by a couple of centrifugation steps (supernatant at 3,000 × g and pellet at 125,000 × g) after homogenization of plant shoots and re-suspended in the buffer (50 mM HEPES-KOH, pH7.8). Microsomal membrane lipids were extract by mixing with 10-fold volume of chloroform: methanol (2: 1, v/v), washed twice with same volume of 0.45% NaCl, and concentrated in chloroform: methanol (2: 1, v/v).

### Pi measurement

Inorganic Pi was extracted separately from shoots and roots, and Pi content was measured using a phosphomolybdate colorimetric assay as described by Chiou et al. (2006). Samples were homogenized with extraction buffer (10 mM Tris, 1.0 mM EDTA, 100 mM NaCl, and 1.0 mM β-mercaptoethanol, pH 8.0). After centrifugation (12,000 × g) for 10 min, 100 μL of supernatant was mixed with 900 μL of 1% glacial acetic acid and incubated at 42°C for 30 min. After centrifugation (120,000 × g) for 5 min, 300 μL of supernatant was mixed with 700 μL of assay solution [0.35% w/v NH_4_MoO_4_, 0.43 M H_2_SO_4_, and 1.4% (w/v) ascorbic acid] and then incubated at 42°C for 30 min. The Pi content was measured at A_820_.

### Measurement of photosynthetic activity

Chlorophyll fluorescence parameters were measured using a Dual-PAM system (Walz). The minimum chlorophyll fluorescence at the open PSII center (Fo) was detected by measuring light (655 nm) at an intensity of 0.05–0.15 μmol m^−2^· s^−1^. A saturating pulse of white light (800 ms) was applied to determine the maximum chlorophyll fluorescence at closed PSII centers in the dark (Fm) and during actinic light illumination (Fm'). Steady-state chlorophyll fluorescence (Fs) was recorded during actinic light illumination (80 μmol photons m^−2^· s^−1^) and Fo' as the minimum chlorophyll fluorescence when actinic light was turned off. *Fv/Fm* was calculated as (Fm - Fo)/Fm. qP was calculated as (Fm' - Fs)/(Fm' - Fo'). NPQ was calculated as (Fm − Fm')/Fm'. ΦII was calculated as (Fm' − Fs)/Fm.

### Measurement of chlorophyll content

Total plant chlorophyll was extracted from homogenized plants using 80% (v/v) acetone. Samples were centrifuged at 12,000 × g at 4°C for 5 min, and then the supernatant was used to measure the absorbance with a spectrophotometer (UV-1600, Shimadzu). Total chlorophyll was calculated using the following formula (Porra et al., 1989):

Total chlorophyll (nmol/mL) = 19.54 ^*^ (A_646.8_ − A_720_) + 8.29 ^*^ (A_663.2_ − A_720_).

### Measurement of sucrose content

Plant seedlings were cut into shoot and root parts and frozen in liquid nitrogen. Soluble sugars were extracted twice in 80% (v/v) ethanol at 80°C for 10 min. Samples were centrifuged at 2,500 × g for 10 min and then dried under N_2_ gas. Glucose content was estimated by using the Glucose Colorimetric/Fluorometric Assay kit (BioVision). To calculate sucrose content, samples were incubated with 50% (v/v) Invertase Solution from Yeast (Wako) at 25°C for 1 h, and the resultant glucose content was estimated as described above.

### Measurement of sucrose uptake

Two-week-old plant seedlings grown on MS medium without sucrose or mannitol were used to estimate sucrose uptake as described by Lei et al. (2011). Briefly, after incubation in MS medium (pH 5.7) for 30 min, the roots were incubated in MS medium containing 0.1% sucrose and [^14^C]sucrose (0.5 mCi·mL^−1^) and incubated for 2 h. After two washes with MS medium containing 1% sucrose, the ^14^C in each seedling was measured in a scintillation counter (LS6500, Beckman) and expressed as cpm·mg^−1^ fresh weight. Each lipid fraction was subjected to thin-layer chromatography (solvent system, acetone: toluene: water = 136: 45: 13), and a radioactive intensity ratio was measured for each fraction using an fluoro-image analyzer (FLA-7000, Fujifilm).

### Conflict of interest statement

The authors declare that the research was conducted in the absence of any commercial or financial relationships that could be construed as a potential conflict of interest.

## References

[B1] Andersson M. X. Larsson K. E. Tjellstrom H. Liljenberg C. Sandelius A. S. (2005). Phosphate-limited oat. The plasma membrane and the tonoplast as major targets for phospholipid-to-glycolipid replacement and stimulation of phospholipases in the plasma membrane. J. Biol. Chem. 280, 27578–27586 10.1074/jbc.M503273200 15927962

[B2] Andersson M. X. Stridh M. H. Larsson K. E. Liljenberg C. Sandelius A. S. (2003). Phosphate-deficient oat replaces a major portion of the plasma membrane phospholipids with the galactolipid digalactosyldiacylglycerol. FEBS Lett. 537, 128–132 10.1016/S0014-5793(03)00109-1 12606044

[B3] Aronsson H. Schöttler M. A. Kelly A. A. Sundqvist C. Dörmann P. Karim S. (2008). Monogalactosyldiacylglycerol deficiency in Arabidopsis affects pigment composition in the prolamellar body and impairs thylakoid membrane energization and photoprotection in leaves. Plant Physiol. 148, 580–592 10.1104/pp.108.123372 18641085 PMC2528128

[B4] Awai K. Maréchal E. Block M. A. Brun D. Masuda T. Shimada H. (2001). Two types of MGDG synthase genes, found widely in both 16:3 and 18:3 plants, differentially mediate galactolipid syntheses in photosynthetic and nonphotosynthetic tissues in *Arabidopsis thaliana*. Proc. Natl. Acad. Sci. U.S.A. 98, 10960–10965 10.1073/pnas.181331498 11553816 PMC58581

[B5] Baena-González E. Rolland F. Thevelein J. M. Sheen J. (2007). A central integrator of transcription networks in plant stress and energy signalling. Nature 448, 938–942 10.1038/nature06069 17671505

[B6] Bari R. Datt Pant B. Stitt M. Scheible W. R. (2006). PHO2, microRNA399, and PHR1 define a phosphate-signaling pathway in plants. Plant Physiol. 141, 988–999 10.1104/pp.106.079707 16679424 PMC1489890

[B7] Bechtold N. Pelletier G. (1998). *In planta* Agrobacterium-mediated transformation of adult *Arabidopsis thaliana* plants by vacuum infiltration. Methods Mol. Biol. 82, 259–266 9664431 10.1385/0-89603-391-0:259

[B8] Benning C. Ohta H. (2005). Three enzyme systems for galactoglycerolipid biosynthesis are coordinately regulated in plants. J. Biol. Chem. 280, 2397–2400 10.1074/jbc.R400032200 15590685

[B9] Bligh E. G. Dyer W. J. (1959). A rapid method of total lipid extraction and purification. Can. J. Biochem. Physiol. 37, 911–917 10.1139/o59-099 13671378

[B10] Block M. A. Dorne A. J. Joyard J. Douce R. (1983). Preparation and characterization of membrane fractions enriched in outer and inner envelope membranes from spinach chloroplasts. II. Biochemical characterization. J. Biol. Chem. 258, 13281–13286 6630230

[B11] Chiou T. J. Aung K. Lin S. I. Wu C. C. Chiang S. F. Su C. L. (2006). Regulation of phosphate homeostasis by MicroRNA in Arabidopsis. Plant Cell 18, 412–421 10.1105/tpc.105.038943 16387831 PMC1356548

[B12] Ciereszko I. Johansson H. Hurry V. Kleczkowski L. A. (2001a). Phosphate status affects the gene expression, protein content and enzymatic activity of UDP-glucose pyrophosphorylase in wild-type and pho mutants of Arabidopsis. Planta 212, 598–605 10.1007/s004250000424 11525517

[B13] Ciereszko I. Johansson H. Kleczkowski L. A. (2001b). Sucrose and light regulation of a cold-inducible UDP-glucose pyrophosphorylase gene via a hexokinase-independent and abscisic acid-insensitive pathway in Arabidopsis. Biochem. J. 354, 67–72 10.1042/0264-6021:3540067 11171080 PMC1221629

[B14] Debast S. Nunes-Nesi A. Hajirezaei M. R. Hofmann J. Sonnewald U. Fernie A. R. (2011). Altering trehalose-6-phosphate content in transgenic potato tubers affects tuber growth and alters responsiveness to hormones during sprouting. Plant Physiol. 156, 1754–1771 10.1104/pp.111.179903 21670224 PMC3149945

[B15] Delatte T. L. Sedijani P. Kondou Y. Matsui M. de Jong G. J. Somsen G. W. (2011). Growth arrest by trehalose-6-phosphate: an astonishing case of primary metabolite control over growth by way of the SnRK1 signaling pathway. Plant Physiol. 157, 160–174 10.1104/pp.111.180422 21753116 PMC3165867

[B16] Douce R. (1974). Site of biosynthesis of galactolipids in spinach chloroplasts. Science 183, 852–853 10.1126/science.183.4127.852 17780772

[B17] Essigmann B. Güler S. Narang R. A. Linke D. Benning C. (1998). Phosphate availability affects the thylakoid lipid composition and the expression of *SQD1*, a gene required for sulfolipid biosynthesis in *Arabidopsis thaliana*. Proc. Natl. Acad. Sci. U.S.A. 95, 1950–1955 10.1073/pnas.95.4.1950 9465123 PMC19220

[B18] Gad M. Awai K. Shimojima M. Yamaryo Y. Shimada H. Masuda T. (2001). Accumulation of plant galactolipid affects cell morphology of *Escherichia coli*. Biochem. Biophys. Res. Commun. 286, 114–118 10.1006/bbrc.2001.5358 11485316

[B19] Gaude N. Nakamura Y. Scheible W. R. Ohta H. Dörmann P. (2008). Phospholipase C5 (NPC5) is involved in galactolipid accumulation during phosphate limitation in leaves of Arabidopsis. Plant J. 56, 28–39 10.1111/j.1365-313X.2008.03582.x 18564386

[B20] Gaude N. Tippmann H. Flemetakis E. Katinakis P. Udvardi M. Dörmann P. (2004). The galactolipid digalactosyldiacylglycerol accumulates in the peribacteroid membrane of nitrogen-fixing nodules of soybean and Lotus. J. Biol. Chem. 279, 34624–34630 10.1074/jbc.M404098200 15159398

[B21] Hammond J. P. Bennett M. J. Bowen H. C. Broadley M. R. Eastwood D. C. May S. T. (2003). Changes in gene expression in Arabidopsis shoots during phosphate starvation and the potential for developing smart plants. Plant Physiol. 132, 578–596 10.1104/pp.103.020941 12805589 PMC166999

[B22] Hammond J. P. White P. J. (2008). Sucrose transport in the phloem: integrating root responses to phosphorus starvation. J. Exp. Bot. 59, 93–109 10.1093/jxb/erm221 18212031

[B23] Härtel H. Benning C. (2000). Can digalactosyldiacylglycerol substitute for phosphatidylcholine upon phosphate deprivation in leaves and roots of Arabidopsis? Biochem. Soc. Trans. 28, 729–732 10.1042/BST0280729 11171187

[B24] Härtel H. Dörmann P. Benning C. (2000). DGD1-independent biosynthesis of extraplastidic galactolipids after phosphate deprivation in *Arabidopsis*. Proc. Natl. Acad. Sci. U.S.A. 97, 10649–10654 10.1073/pnas.180320497 10973486 PMC27079

[B25] Jarvis P. Dörmann P. Peto C. A. Lutes J. Benning C. Chory J. (2000). Galactolipid deficiency and abnormal chloroplast development in the *Arabidopsis MGD synthase 1* mutant. Proc. Natl. Acad. Sci. U.S.A. 97, 8175–8179 10.1073/pnas.100132197 10869420 PMC16689

[B26] Jouhet J. Maréchal E. Baldan B. Bligny R. Joyard J. Block M. A. (2004). Phosphate deprivation induces transfer of DGDG galactolipid from chloroplast to mitochondria. J. Cell Biol. 167, 863–874 10.1083/jcb.200407022 15569715 PMC2172463

[B27] Joyard J. Maréchal E. Miège C. Block M. A. Dorne A. J. Douce R. (1998). Structure, distribution and biosynthesis of glycerolipids from higher plant chloroplasts, in Lipid in Photosynthesis: Structure, Function and Genetics, eds Siegenthaler P. A. Murata N. (Dordrecht: Kluwer Academic Publishers), 21–52

[B28] Karthikeyan A. S. Varadarajan D. K. Jain A. Held M. A. Carpita N. C. Raghothama K. G. (2007). Phosphate starvation responses are mediated by sugar signaling in Arabidopsis. Planta 225, 907–918 10.1007/s00425-006-0408-8 17033812

[B29] Kelly A. A. Dörmann P. (2002). *DGD2*, an *Arabidopsis* gene encoding a UDP-galactose-dependent digalactosyldiacylglycerol synthase is expressed during growth under phosphate-limiting conditions. J. Biol. Chem. 277, 1166–1173 10.1074/jbc.M110066200 11696551

[B30] Kelly A. A. Froehlich J. E. Dörmann P. (2003). Disruption of the two digalactosyldiacylglycerol synthase genes *DGD1* and *DGD2* in Arabidopsis reveals the existence of an additional enzyme of galactolipid synthesis. Plant Cell 15, 2694–2706 10.1105/tpc.016675 14600212 PMC280572

[B31] Kobayashi K. Awai K. Nakamura M. Nagatani A. Masuda T. Ohta H. (2009a). Type-B monogalactosyldiacylglycerol synthases are involved in phosphate starvation-induced lipid remodeling, and are crucial for low-phosphate adaptation. Plant J. 57, 322–331 10.1111/j.1365-313X.2008.03692.x 18808455

[B33] Kobayashi K. Awai K. Takamiya K. Ohta H. (2004). Arabidopsis type B monogalactosyldiacylglycerol synthase genes are expressed during pollen tube growth and induced by phosphate starvation. Plant Physiol. 134, 640–648 10.1104/pp.103.032656 14730084 PMC344540

[B34] Kobayashi K. Kondo M. Fukuda H. Nishimura M. Ohta H. (2007). Galactolipid synthesis in chloroplast inner envelope is essential for proper thylakoid biogenesis, photosynthesis, and embryogenesis. Proc. Natl. Acad. Sci. U.S.A. 104, 17216–17221 10.1073/pnas.0704680104 17940034 PMC2040463

[B35] Kobayashi K. Masuda T. Takamiya K. Ohta H. (2006). Membrane lipid alteration during phosphate starvation is regulated by phosphate signaling and auxin/cytokinin cross-talk. Plant J. 47, 238–248 10.1111/j.1365-313X.2006.02778.x 16762032

[B32] Kobayashi K. Nakamura Y. Ohta H. (2009b). Type A and type B monogalactosyldiacylglycerol synthases are spatially and functionally separated in the plastids of higher plants. Plant Physiol. Biochem. 47, 518–525 10.1016/j.plaphy.2008.12.012 19179086

[B36] Kobayashi K. Narise T. Sonoike K. Hashimoto H. Sato N. Kondo M. (2012). Role of galactolipid biosynthesis in coordinated development of photosynthetic complexes and thylakoid membranes during chloroplast biogenesis in Arabidopsis. Plant J. 73, 250–261 10.1111/tpj.12028 22978702

[B37] Kochian L. V. (2012). Plant nutrition: rooting for more phosphorus. Nature 488, 466–467 10.1038/488466a 22914160

[B38] Lambers H. Cawthray G. R. Giavalisco P. Kuo J. Laliberté E. Pearse S. J. (2012). Proteaceae from severely phosphorus-impoverished soils extensively replace phospholipids with galactolipids and sulfolipids during leaf development to achieve a high photosynthetic phosphorus-use-efficiency. New Phytol. 196, 1098–1108 10.1111/j.1469-8137.2012.04285.x 22937909

[B39] Lei M. Liu D. (2011). Sucrose regulates plant responses to deficiencies in multiple nutrients. Plant Signal. Behav. 6, 1247–1249 10.4161/psb.6.8.16378 21701258 PMC3260736

[B40] Lei M. Liu Y. Zhang B. Zhao Y. Wang X. Zhou Y. (2011). Genetic and genomic evidence that sucrose is a global regulator of plant responses to phosphate starvation in Arabidopsis. Plant Physiol. 156, 1116–1130 10.1104/pp.110.171736 21346170 PMC3135933

[B41] Lejay L. Gansel X. Cerezo M. Tillard P. Muller C. Krapp A. (2003). Regulation of root ion transporters by photosynthesis: functional importance and relation with hexokinase. Plant Cell. 15, 2218–2232 10.1105/tpc.013516 12953122 PMC181342

[B42] Lunn J. E. Feil R. Hendriks J. H. Gibon Y. Morcuende R. Osuna D. (2006). Sugar-induced increases in trehalose 6-phosphate are correlated with redox activation of ADPglucose pyrophosphorylase and higher rates of starch synthesis in *Arabidopsis thaliana*. Biochem. J 397, 139–148 10.1042/BJ20060083 16551270 PMC1479759

[B43] Lynch J. P. (2011). Root phenes for enhanced soil exploration and phosphorus acquisition: tools for future crops. Plant Physiol. 156, 1041–1049 10.1104/pp.111.175414 21610180 PMC3135935

[B44] Martín A. C. del Pozo J. C. Iglesias J. Rubio V. Solano R. de La Peña A. (2000). Influence of cytokinins on the expression of phosphate starvation responsive genes in *Arabidopsis*. Plant J. 24, 559–567 10.1046/j.1365-313x.2000.00893.x 11123795

[B45] Martínez-Barajas E. Delatte T. Schluepmann H. de Jong G. J. Somsen G. W. Nunes C. (2011). Wheat grain development is characterized by remarkable trehalose 6-phosphate accumulation pregrain filling: tissue distribution and relationship to SNF1-related protein kinase1 activity. Plant Physiol. 156, 373–381 10.1104/pp.111.174524 21402798 PMC3091070

[B46] Misson J. Raghothama K. G. Jain A. Jouhet J. Block M. A. Bligny R. (2005). A genome-wide transcriptional analysis using *Arabidopsis thaliana* Affymetrix gene chips determined plant responses to phosphate deprivation. Proc. Natl. Acad. Sci. U.S.A. 102, 11934–11939 10.1073/pnas.0505266102 16085708 PMC1188001

[B47] Müller R. Morant M. Jarmer H. Nilsson L. Nielsen T. H. (2007). Genome-wide analysis of the Arabidopsis leaf transcriptome reveals interaction of phosphate and sugar metabolism. Plant Physiol. 143, 156–171 10.1104/pp.106.090167 17085508 PMC1761981

[B48] Müller R. Nilsson L. Nielsen L. K. Nielsen T. H. (2005). Interaction between phosphate starvation signalling and hexokinase-independent sugar sensing in *Arabidopsis* leaves. Physiol. Plant 124, 81–90 10.1111/j.1399-3054.2005.00496.x

[B49] Murashige T. Skoog F. (1962). A revised medium for rapid growth and bioassays with tobacco tissue cultures. Physiol. Plant 15, 473–497 10.1111/j.1399-3054.1962.tb08052.x

[B50] Murphy D. J. (1986). The molecular organisation of the photosynthetic membranes of higher plants. Biochim. Biophys. Acta 864, 33–94 10.1016/0304-4157(86)90015-8

[B51] Nakamura Y. (2013). Phosphate starvation and membrane lipid remodeling in seed plants. Prog. Lipid Res. 52, 43–50 10.1016/j.plipres.2012.07.002 22954597

[B52] Narise T. Kobayashi K. Baba S. Shimojima M. Masuda S. Fukaki H. (2010). Involvement of auxin signaling mediated by IAA14 and ARF7/19 in membrane lipid remodeling during phosphate starvation. Plant Mol. Biol. 72, 533–544 10.1007/s11103-009-9589-4 20043234

[B53] Nielsen T. H. Krapp A. Röper-Schwarz U. Stitt M. (1998). The sugar-mediated regulation of genes encoding the small subunit of Rubisco and the regulatory subunit of ADP glucose pyrophosphorylase is modified by phosphate and nitrogen. Plant Cell Environ. 21, 443–454 10.1046/j.1365-3040.1998.00295.x

[B54] Nunes C. Primavesi L. F. Patel M. K. Martinez-Barajas E. Powers S. J. Sagar R. (2013). Inhibition of SnRK1 by metabolites: tissue-dependent effects and cooperative inhibition by glucose 1-phosphate in combination with trehalose 6-phosphate. Plant Physiol. Biochem. 63, 89–98 10.1016/j.plaphy.2012.11.011 23257075

[B55] Ohta H. Yuzawa Y. Shimojima M. (2012). Galactoglycerolipids, in ASBMB Today August, 28–29 Available online at: http://www.asbmb.org/asbmbtoday/asbmbtoday_article.aspx?id=17476

[B55a] Porra R. J. Thompson W. A. Kriedemann P. E. (1989). Determination of accurate extinction coefficients and simultaneous equations for assaying chlorophylls a and b extracted with four different solvents: verification of the concentration of chlorophyll standards by atomic absorption spectroscopy. Biochim. Biophys. Acta 975, 384–394 10.1016/S0005-2728(89)80347-0

[B56] Rubio V. Linhares F. Solano R. Martín A. C. Iglesias J. Leyva A. (2001). A conserved MYB transcription factor involved in phosphate starvation signaling both in vascular plants and in unicellular algae. Genes Dev. 15, 2122–2133 10.1101/gad.204401 11511543 PMC312755

[B57] Russo M. A. Quartacci M. F. Izzo R. Belligno A. Navari-Izzo F. (2007). Long- and short-term phosphate deprivation in bean roots: plasma membrane lipid alterations and transient stimulation of phospholipases. Phytochemistry 68, 1564–1571 10.1016/j.phytochem.2007.03.017 17466344

[B58] Sanz L. Dewitte W. Forzani C. Patell F. Nieuwland J. Wen B. (2011). The Arabidopsis D-Type Cyclin CYCD2;1 and the Inhibitor ICK2/KRP2 Modulate Auxin-Induced Lateral Root Formation. Plant Cell 23, 641–660 10.1105/tpc.110.080002 21357490 PMC3077792

[B59] Schluepmann H. van Dijken A. Aghdasi M. Wobbes B. Paul M. Smeekens S. (2004). Trehalose mediated growth inhibition of Arabidopsis seedlings is due to trehalose-6-phosphate accumulation. Plant Physiol. 135, 879–890 10.1104/pp.104.039503 15181209 PMC514123

[B60] Shimojima M. Ohta H. Iwamatsu A. Masuda T. Shioi Y. Takamiya K. (1997). Cloning of the gene for monogalactosyldiacylglycerol synthase and its evolutionary origin. Proc. Natl. Acad. Sci. U.S.A. 94, 333–337 10.1073/pnas.94.1.333 8990209 PMC19336

[B61] Shimojima M. Watanabe T. Madoka Y. Koizumi R. Yamamoto M. P. Masuda K. (2013). Differential regulation of two types of monogalactosyldiacylglycerol synthase in membrane lipid remodeling under phosphate-limited conditions in sesame plants. Front. Plant Sci. 4:469 10.3389/fpls.2013.00469 24312111 PMC3832787

[B62] Tjellström H. Andersson M. X. Larsson K. E. Sandelius A. S. (2008). Membrane phospholipids as a phosphate reserve: the dynamic nature of phospholipid-to-digalactosyl diacylglycerol exchange in higher plants. Plant Cell Environ. 31, 1388–1398 10.1111/j.1365-3040.2008.01851.x 18643953

[B63] Vance C. P. Uhde-Stone C. Allan D. L. (2003). Phosphorus acquisition and use: critical adaptation by plants for securing a nonrenewable resource. New Phytol. 157, 423–447 10.1046/j.1469-8137.2003.00695.x 33873400

[B64] Wu P. Ma L. Hou X. Wang M. Wu Y. Liu F. (2003). Phosphate starvation triggers distinct alterations of genome expression in Arabidopsis roots and leaves. Plant Physiol. 132, 1260–1271 10.1104/pp.103.021022 12857808 PMC167066

[B65] Xu C. Fan J. Froehlich J. E. Awai K. Benning C. (2005). Mutation of the TGD1 chloroplast envelope protein affects phosphatidate metabolism in Arabidopsis. Plant Cell 17, 3094–3110 10.1105/tpc.105.035592 16199613 PMC1276032

[B66] Yamaryo Y. Kanai D. Awai K. Shimojima M. Masuda T. Shimada H. (2003). Light and cytokinin play a co-operative role in MGDG synthesis in greening cucumber cotyledons. Plant Cell Physiol. 44, 844–855 10.1093/pcp/pcg110 12941877

[B67] Yuzawa Y. Nishihara H. Haraguchi T. Masuda S. Shimojima M. Shimoyama A. (2012). Phylogeny of galactolipid synthase homologs together with their enzymatic analyses revealed a possible origin and divergence time for photosynthetic membrane biogenesis. DNA Res. 19, 91–102 10.1093/dnares/dsr044 22210603 PMC3276260

[B68] Zhang Y. Primavesi L. F. Jhurreea D. Andralojc P. J. Mitchell R. A. Powers S. J. (2009). Inhibition of SNF1-related protein kinase1 activity and regulation of metabolic pathways by trehalose-6-phosphate. Plant Physiol. 149, 1860–1871 10.1104/pp.108.133934 19193861 PMC2663748

